# Spatial distance between anatomically- and physiologically-identified targets in subthalamic nucleus deep brain stimulation in Parkinson’s disease

**Published:** 2016-01-05

**Authors:** Mansour Parvaresh-Rizi, Alireza Tabibkhoei, Gholamali Shahidi, Janardan Vaidyanathan, Amirreza Tabibkhoei, Mohammad Rohani

**Affiliations:** 1 Department of Neurosurgery, School of Medicine AND Rasoul-E-Akram Hospital, Iran University of Medical Sciences, Tehran, Iran; 2 Neuromodulation, ‎Medtronic, Mumbai, India

**Keywords:** Parkinson's Disease, Deep Brain Stimulation, Intraoperative Monitoring, Anatomic Target, Physiologic Target

## Abstract

**Background:** Subthalamic nucleus (STN) stimulation is the treatment of choice for carefully chosen patients with idiopathic Parkinson's disease (PD) and refractory motor fluctuations. We evaluated the value of intraoperative electrophysiology during STN deep brain stimulation (DBS) procedures in refining the anatomically-defined target.

**Methods:** We determined the spatial distance between the anatomical and physiological targets along x, y and z axes in 50 patients with PD who underwent bilateral subthalamic nucleus DBS surgery.

**Results:** The mean spatial distance between anatomical and functional targets was 1.84 ± 0.88 mm and the least distances in different methods were 0.66 mm [standard error (SE): 0.07], 1.07 mm (SE: 0.08) and 1.01 mm (SE: 0.08) on x, y and z axes, respectively, for the combined method.

**Conclusion:** The most physiologically-accurate anatomical targeting was achieved via a combination of multiple independent methods. There was a statistically significant difference between the anatomical and functional targets in all methods (even the combined) on the y coordinate, emphasizing the need for intra-operative electrophysiological monitoring to refine the anatomico-radiologically-defined target.

## Introduction

Subthalamic nucleus (STN) stimulation is an effective therapy for the amelioration of Parkinson’s disease (PD) motor symptomatology and drug-induced dyskinesias.^1^^-^^7^

STN deep brain stimulation (DBS) is the surgical treatment of choice for medically refractory PD in carefully selected patients.^6^^,^^8^^,^^9^ However, the best means of targeting this nucleus still remains a matter of discussion. This is partly because of the small size of the STN, its biconvex shape, and triple oblique orientation.^8^^,^^10^ Due to lack of contrast between the STN and surrounding structures on regular computed tomography (CT) and T1 weighted magnetic resonance imaging (MRI) sequences, information from these modalities are often complemented with T2 weighted MR images, printed and digitalized anatomical brain atlases, high-resolution T1-T2 maps, functional atlases, and databases. In addition, integration of multiple functional and anatomical references may also be employed to facilitate surgical targeting.^11^

The accuracy of DBS lead placement and electrode location planning is the key factor for therapeutic efficacy.^4^^,^^10^^,^^12^^,^^13^ A small deviation in the electrode positioning may cause severe side effects such as speech disorders, muscle contractions, ocular deviations, or visual defects to name a few. Hence, it is critical to perform precise surgical target localization, reduce error at every stage of the procedure and perform electrode location planning to achieve optimal surgical outcomes.

The specific objectives of this cross-sectional retrospective study were to identify the distance between the anatomical and functional target based on each targeting method, evaluate the confidence provided by each anatomical targeting method and defining the spatial position of the functional target without performing post-operative conditionally safe MRI scans.

## Materials and Methods

This retrospective cross-sectional study included data from 50 idiopathic PD patients referred to our department (41 males and 9 females) from July 2006 to September 2009, aged between 31 and 72 years for bilateral STN-DBS (a total of 100 procedures). Each patient was carefully selected by a team of specialists consisting of a movement disorder neurologist, a functional stereotactic neurosurgeon, psychologist and a neuropsychiatric.

Patients were selected as per the following criteria: age under 75 years, disabling motor fluctuations and drug-induced dyskinesia refractory to medical therapy. The exclusion criteria were as follows: the presence of cognitive impairment, major depression or marked cerebral (both cortical and ventricular) atrophy on neuroimaging studies.

**Figure 1:**
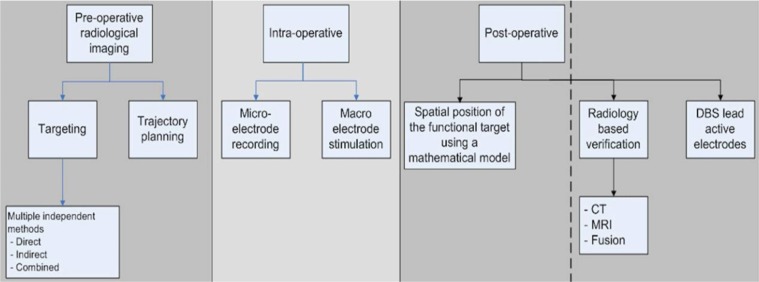
Scope of the study

The scope of the study is as shown in figure 1. It includes the pre-operative targeting based on multiple independent methods (direct, indirect and combined methods) which are compared to the intra-operative results of microelectrode recording (MER) and macroelectrode stimulation (MES). Postoperatively the spatial position of the functional target is calculated using a mathematical model without performing a post-operative MRI.

***Acquisition of image data***

Placement of the Leksell G stereotactic frame (Elekta Instruments AB, Stockholm, Sweden) with an attached MRI compatible localizer was performed prior to MRI for each patient. The frame was placed parallel to the orbitomeatal line using ear bars inserted into the patient’s external auditory meatus that were attached to the base ring and then pivoting the base ring into the desired alignment.

General anesthesia was maintained during imaging and with the head frame fixed within the head coil. The patient remained immobilized during the MRI acquisition reducing potential movement-related artifacts. The following stereotactic brain MRI sequences were obtained using a 1.5 tesla Philips Gyroscan MRI scanner:

- Pre-operative three-dimensional (3D) T1 weighted volumetric sequence with an isotropic voxel (1 × 1 × 1 mm) acquired using an intravenous contrast to enhance the definition of blood vessels

- Two sets of coronally and axially oriented two-dimensional T2 weighted fast spin echo sequences (TR: 2800 ms, TE: 110 ms, flip angle 90°, NEX 4, square pixel of size: 1 × 1 mm, slice thickness 1.5 mm, inter-slice spacing 0 mm, matrix 256 × 256).

None of the above sequences had gantry angulation (zero tilt), and the sequences were strictly axial or coronal. The radiology department shimmed the magnets on a regular basis to minimize the distortion in MRI sequences.

***Anatomical targeting***

The coordinates were calculated with respect to the Leksell stereotactic system with the arc used in the lateral right configuration. No fusion was used in this approach to minimize the error of the fusion algorithms. The following six independent anatomical targeting methods were used:

Atlas-based targeting: We used the digitalized version of the Schaltenbrand and Wahren stereotactic brain atlas reformatted and linearly scaled to the anterior commissure-posterior commissure to fit the individual patient’s anatomy (e.g., width of the third ventricle). The center of the motor part of STN on each side was chosen and its coordinates were determined.^14^

T2 weighted MRI-based direct targeting with manual calculations on axial and coronal orientations: The STN is located lateral to the red nucleus (RN), dorsal to the substantia nigra (SN) and medial to the posterior limb of the internal capsule, has a hypointense signal intensity on T2 weighted MRI. The anterior and lateral boundaries of the RN can be best visualized on an axial T2 weighted MRI. The anatomical relationship between these three structures can assist in the identification of the surgical target location within the STN. We used the axial and coronal planes of T2 weighted images separately to define the dorsolateral part of the STN for targeting and two sets of coordinates (X, Y, and Z) were determined. Calculations on the axial and coronal plane were done manually on the MRI console.

T2 weighted MRI-based targeting by Stereonauta software: Using above relationships between the three structures (SN, RN, and STN), after registration of the stereotactic images on the Stereonauta software (Estudios e Investigaciones Neurológicas, S.L., Madrid, Spain), we determined the target on axial and coronal T2 weighted images separately. Two sets of coordinates (X, Y, and Z) were determined. In all the 50 patients, we considered a mean error of < 0.5 mm for registration of the Leksell stereotactic frame in Stereonauta software.

Combined method: The last set of coordinates was a combination of the aforementioned five methods, which was defined by the stereotactic neurosurgeon as an average of all previous coordinates, heuristically considering outliers. 

After defining the anatomical target, the safest trajectory for electrophysiological exploration (with five simultaneous trajectories) was determined on the 3D T1 weighted contrast enhanced MRI with a 6 mm circle of safety. A look ahead up to 10 mm beyond the target was done to reaffirm safety. These trajectory settings were arc and ring angles on the Leksell stereotactic system with the arc used in the lateral right configuration.

***Surgical procedure***

All patients gave their informed consent. Antiparkinsonian medications were withdrawn the night before surgery (the long acting medications withdrawn earlier as deemed appropriate by the movement disorder neurologist). Under general anesthesia, in semi sitting position the stereotactic frame was fixed, imaging and targeting performed and then attached to the Mayfield head holder. Under strict sterile conditions, a C-shaped incision on the coronal suture was made after marking the entry points on the skin with the stereotactic guidance and a cutaneous flap reflected. Two 14 mm burr holes were made on the uppermost part of the cranium according to planned trajectory about 4 cm from the midline and anterior to the coronal suture. The dura was opened in a circular shape of about 5 mm in diameter first on the left side. After completion of the procedure on the left side, the dura was opened in a similar manner on the right side. Continuous irrigation with normal saline minimized entry of intracranial air and possible brain shift.

Using a stereotactic micro-drive (microTargeting drive, FHC Inc., Bowdoin, MI, USA) five parallel platinum-iridium microelectrodes were inserted through the dural opening directed to the location of the combined anatomical target. These five electrodes were arranged in a “+ plus” configuration resulting in central, anterior posterior, medial and lateral parallel trajectories. The distance between the central trajectory to others was 2 mm, measured center to center. MER was started 10 mm above the anatomical target and continued in incremental steps of 0.5 mm, and the discharge pattern of neurons was identified. Below the thalamus we usually found some cells with a low firing rate that probably belong to a thin strip of gray matter located between the thalamic and lenticular fasciculi, the zona incerta. After this, a marked increase in the background noise defined the STN which cells have large amplitudes and an irregular firing pattern with a firing rate of around 25-50 Hz. Finally, without a clear border the electrodes entered the SN with low background noise and high-frequency tonic discharge.

The length of MER recordings along each trajectory was determined, and a 3D electrophysiological view of the STN was inferred. After that the recording electrodes, were withdrawn by 10 mm and the overlying macroelectrode in the selected trajectory was introduced (other macroelectrodes remained withdrawn to prevent a possible microsubthalamotomy effect). The therapeutic window (the difference between the intensity of electrical current thresholds of best clinical effects and side-effects) predicts clinical long-term efficacy and determines which trajectory and to which point along this trajectory, the permanent DBS lead should be implanted for optimal clinical results. This point is our gold standard (the physiological/functional target) according to which, the accuracy of other anatomical targeting methods could be assessed. The permanent DBS lead contains four electrodes, centered on this functional target each measuring 1.5 mm in width with 0.5 mm spacing in between them and a diameter of 1.27 mm (model-3389, Medtronic Inc., Minneapolis, USA).

Using propofol and dexmedetomidine for maintenance of general anesthesia with bispectral index (BIS) monitoring we could lighten the patients just before initiation of recording and stimulation. Clinical effects of stimulation were monitored with contralateral hand tremor, wrist rigidity, and bradykinesia. The beneficial effects of acute test stimulation were observed in the 1-3 mAmp range. Side effects secondary to stimulation included contralateral muscle contractions and/or eye deviation among others as mentioned in literature. MES up to a supramaximal threshold of 5 mAmps was considered acceptable to choose a trajectory for implanting the DBS lead with intra-operative fluoroscopic guidance using lateral crosshairs. A synopsis of side-effects of acute macro-stimulation in the STN region are ‎summarized in table 1.

The day after both DBS leads were placed we implanted the implantable neurostimulator (model Kinetra, Medtronic Inc., Minneapolis, USA) in the left infraclavicular area subcutaneously. All patients underwent a post-operative brain CT scan to rule out possible complications especially intracerebral hemorrhage (ICH). We followed them for a minimum of 1 year for the onset of delayed complications such as infection.

***Calculation of target deviation***

**Figure 2:**
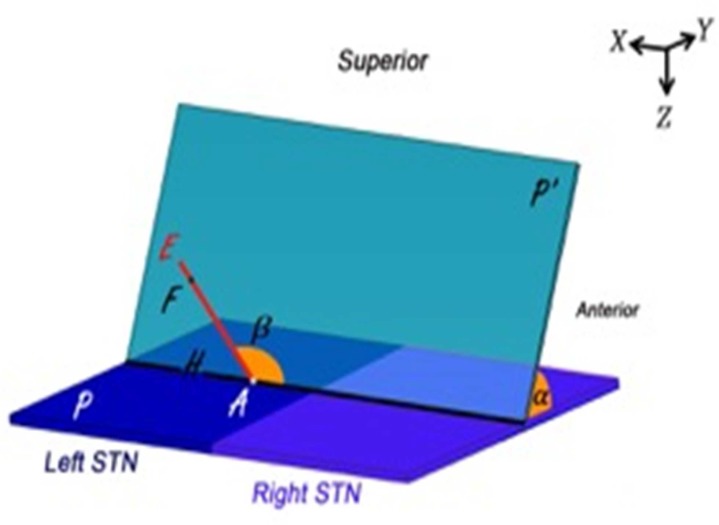
Schematic for calculation of target deviation

As depicted in figure 2, plane P is the axial plane through combined anatomical point (A). Plane P' is the plane of “arc” (=) and line AE is the central trajectory in this plane defined with “ring” angle =  . Point F is the functional target along the trajectory AE.

According to the angle between plane P' and plane P, normal vector of P' is:


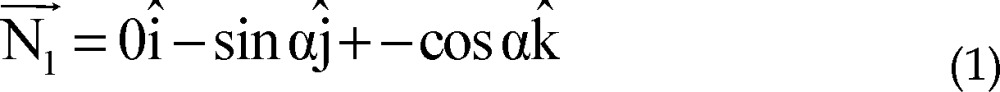


Since point A is in the plane P' and its coordinates are defined, the equation of plane p' could be expressed as:


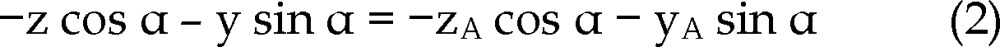


Now, by equating dot product $\overset{\text{⃗}}{\mathrm{AE}}.\overset{\text{⃗}}{\mathrm{AH}}(\left|\overset{\text{⃗}}{\mathrm{AH}}\right|=1)$ with cosine of the angle between them:


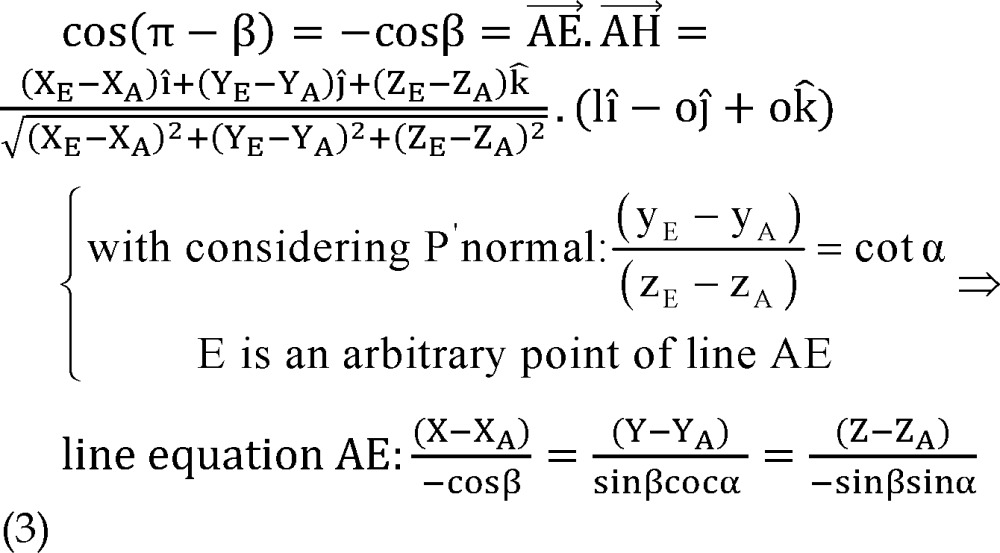


And direction vector of line AE can be also derived:


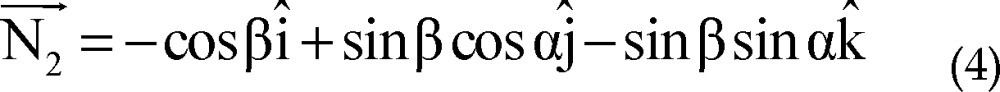


**Figure 3:**
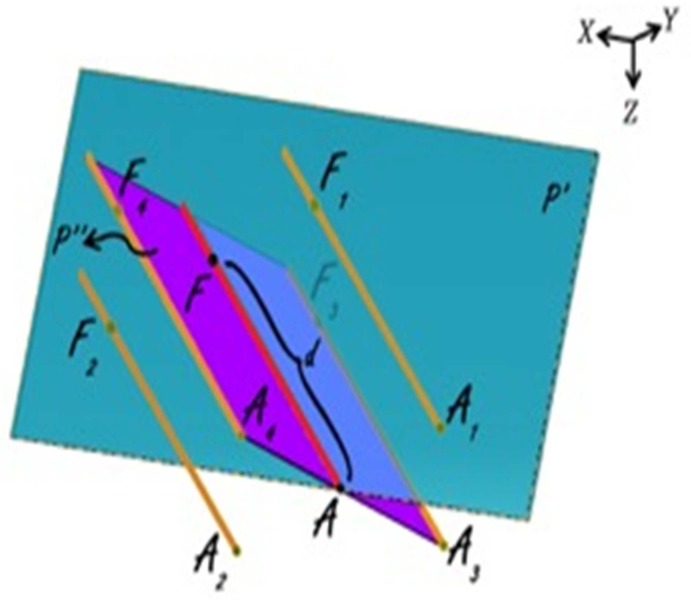
Anatomical and functional targets

As portrayed in figure 3 points A_1_, A_2_, A_3_ and A_4_ are respective to point A on other trajectories at the same level on the microdriver and the functional target could be also on trajectories other than central (points F_1_, F_2_, F_3_ and F_4_) with defined distance to points A_i_ (d). The functional target may be inferior than the anatomical target (−d), which is not shown in figure 3.

**Table 1:** The side-effects of synopsis acute macro-stimulation in the subthalamic nucleus ‎region ‎for Parkinson's disease (PD)

**Motor**	**Occulomotor**	**Sensory**	**Autonomic**	**Affective**	**Limbic**
Tonic at high frequency and tetanic at low frequency, used to distinguish between off-phase and stimulation-induced dystonia Evoked by current spreading to the corticobulbar and corticospinal tracts which surround the STN anterolaterally passing through the posterior limb of the internal capsule No habituation, consider a more medial or posterior trajectory if the threshold is very low resulting in a narrow therapeutic window Usually present as tonic contraction and phasic fasciculation contralaterally or bilaterally to stimulation in the face (forehead, eyebrow, eyelid muscles, cheek, lip or chin muscles), contractions of the contralateral upper limb intrinsic muscles more than the lower limb Dysarthia and dysphonia usually time locked with stimulation	**Extrinsic** Ipsilateral eye adduction, upward or downward deviation or lid retraction Side effects related to current spread to third cranial nerve fibers which pass ventromedially to the STN, close to the posterior border of the red nucleus and to the medial part of the substantia nigra, before leaving the brainstem Usually do not habituate If these occur at low or medium stimulation intensities consider a more lateral trajectory for evaluation Reduced voluntary ipsilateral conjugate eye deviation progressively resulting in conjugate controversive eye deviation (to the stimulation side) at higher intensities are related to stimulation of the occulomotor corticoganglionic loop inside the STN or the activation of the prefrontal-occulomotor projections to diecephalic and brainstem structures in the lateral part of the STN. Stimulation-induced conjugate ocular deviation rapidly habituates and even if elicited at low-intensity thresholds does not imply the need to explore another trajectory. **Intrinsic** Bilateral asymmetric mydriasis more marked for the ipsilateral side of stimulation can be induced by stimulation of the descending sympathetic fibers in the zona incerta (dorsomedially to the STN) or by stimulation of posteroventral hypothalamus, the so-called sympathetic hypothalamic area of Hess (anteriorly to the STN) These symptoms are rapidly adapting and usually these side-effects do not require a change in trajectory. Unilateral change in pupil diameter, homolateral to the stimulation side with or without an ipsilateral eye deviation occurs with the stimulation of the parasympathetic component of the third cranial nerve If the above occurs at low stimulation intensities a lateral trajectory has to be considered (as the trajectory may be located too medially)	Contralateral hemibody transient parasthesias could be the sensory-motor part of STN Dysesthesias at low stimulation intensities in the upper or lower limb could be due to stimulation of the red nucleus (which is more medial and posterior). Consider an anteriolateral trajectory Persistent parasthesias could be due to current spreading to the medial leminscus located posterioventral to the STN	Heat sensation, flushing, sweating, piloerection, nausea, vomiting, vasoconstriction, changes in hemodynamics Could be because of current spreading to the limbic part of STN or to the descending sympathetic fibers passing in the zona incerta (dorsomedial to the STN) or to the posterior part of the hypothalamus (anterior to the STN) These symptoms do not persist in the long term Stimulation site acceptable if there are optimal benefits on Parkinson’s symptoms	Feeling electric current initially, dizziness, anxiety, breathing difficulties, discomfort in the chest, uncomfortable feeling in the head or with vision These side-effects cannot be related specifically to any anatomical sub-structure Usually tolerate and the trajectory can be used if there is optimal benefit on Parkinson’s symptoms	Euphoria, hypomania or acute depressive states may occur time locked with stimulation rarely Pathologic or mirthful laughter or pseudobulbar crying May occur due to current spreading in the limbic part of STN or substantia nigra

STN: Subthalamic nucleus

According to figure 3, plane P'' is orthogonal to plane P' and AF is the intersection of these two planes.

By using the cross product of direction vector of line AF and normal vector of plane P', the normal vector of plane P'' would be determined as:


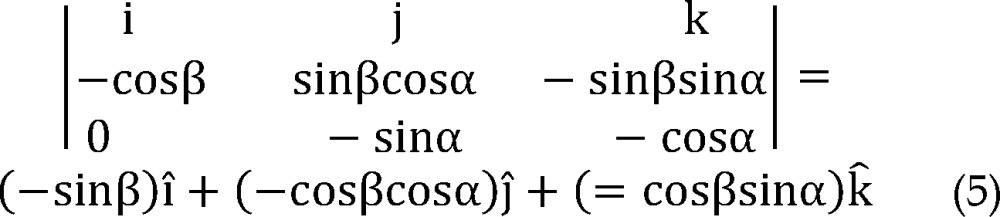


By using normal vector of plane P'', equation of plane P'' passing through point A could be expressed as:


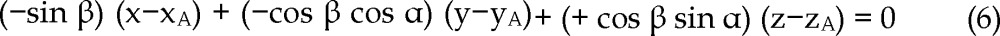


Coordinates of points A_1_ and A_2_ are considered as:


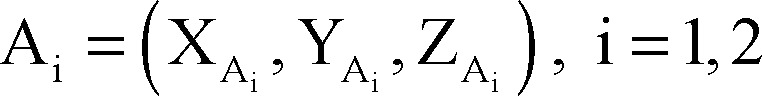


The distance between points A_1_ and A_2_ to point A is 2 mm, so,


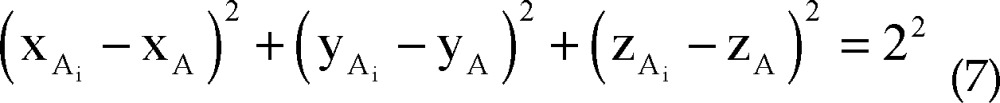


Points A, A_1_, A_2_, A_3_ and A_4_ are on the same plane. 

Plane P' passes through points A_1_ and A_2_, thus:


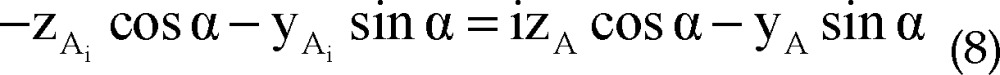


Direction vector of line AA_i_ is perpendicular to normal vector of line AF:


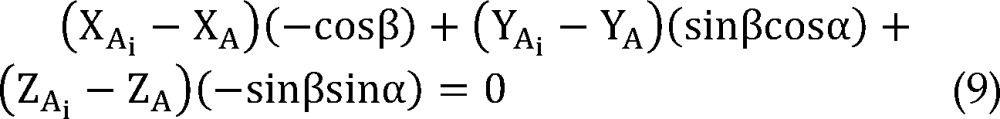


By solving simultaneous equations 7, 8 and 9, parametric values of A_1_ and A_2_ could be expressed as:


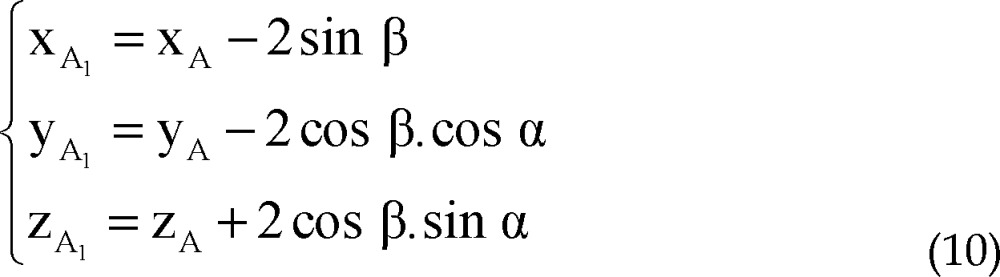



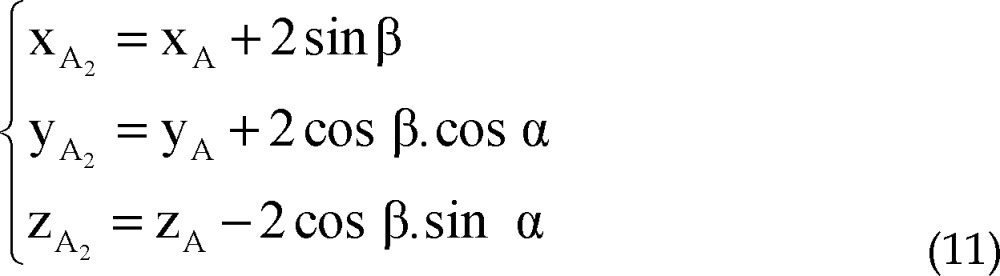


Coordinates of points A_3_ and A_4_ are considered as:


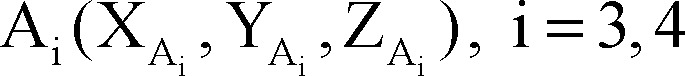


The distance between points A_3_ and A_4_ to point A is 2 mm, plane P'' passes through points A_3_ and A_4_ and direction vector of line AA_i_ is perpendicular to normal vector of line AF.

Similarly to A_1_, A_2_, parametric values of A_3_, A_4_ could be expressed as:


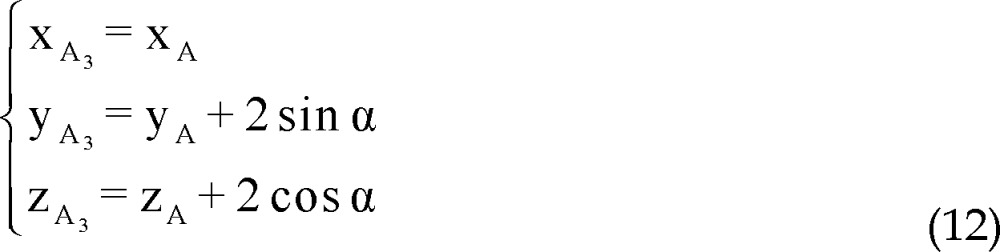



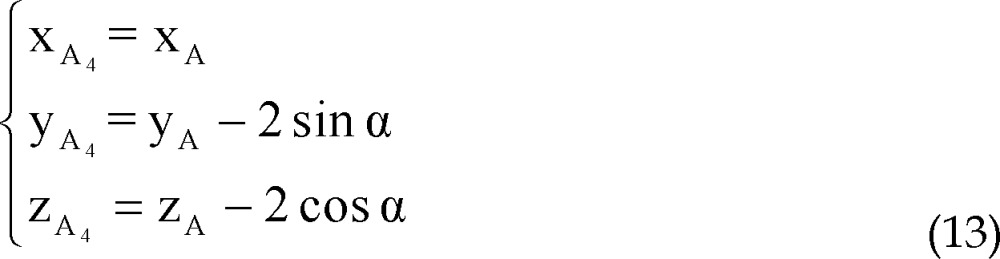


Note that direction vectors of all trajectories A_i_F_i_ (i = 1, 2, 3, 4) are equal to direction vector of line AF and their length is 1 mm and also all points F_i_ (i = 1, 2, 3, 4) are on the respective lines A_i_F_i_ (i = 1, 2, 3, 4), so to clarify coordinates of points F_i_:


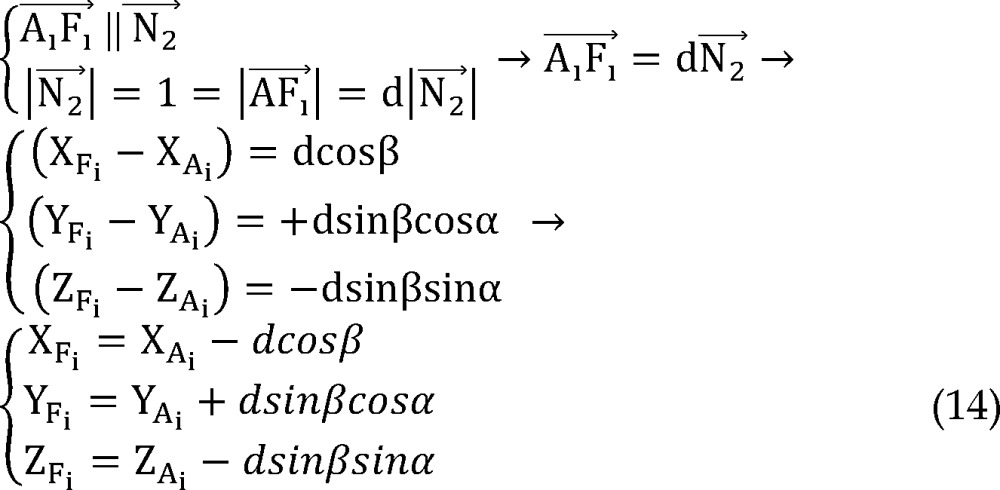


Using all of equations 10, 11, 12 and 13 in equation 14 we determined parametric values of F_1_, F_2_, F_3_ and F_4_ respectively:

Right lateral trajectory and left medial trajectory:


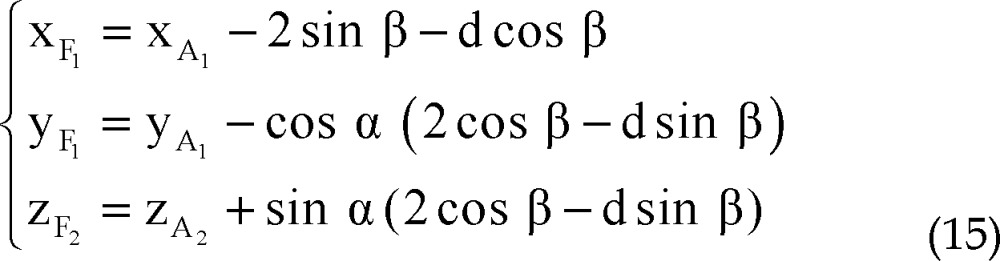


Right medial trajectory and left lateral trajectory:


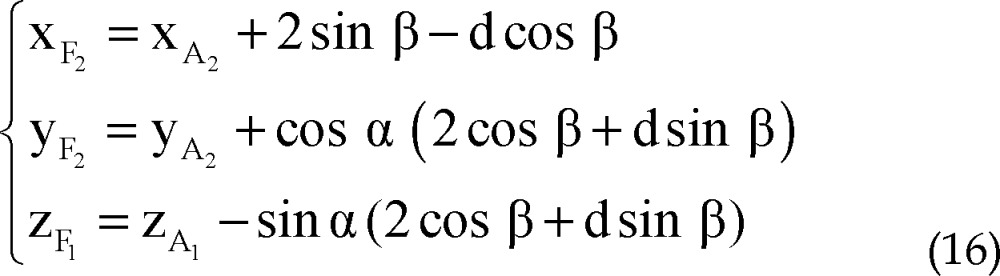


Right or left anterior trajectory:


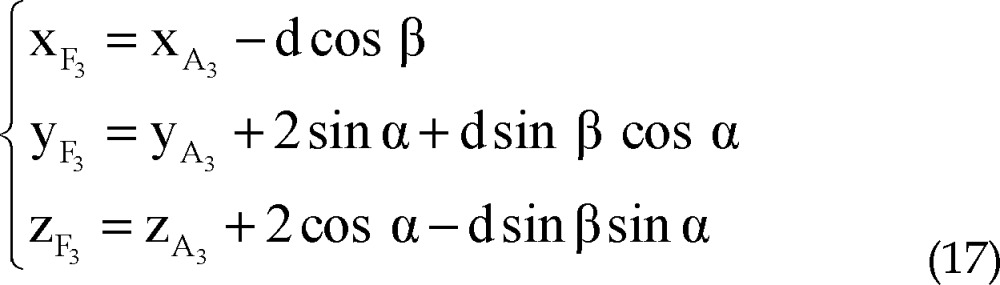


Right or left posterior trajectory:


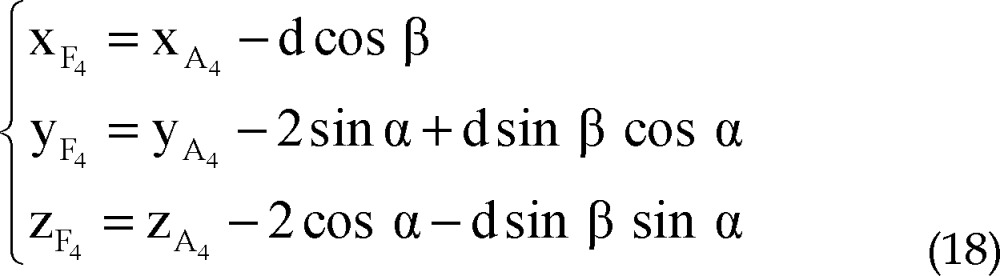


Right or left central trajectory:


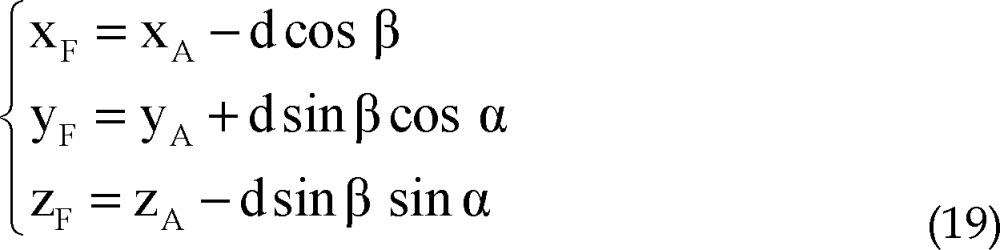


Finally the spatial distance between anatomical (A) and functional (F_i_) targets (delta) was calculated as:


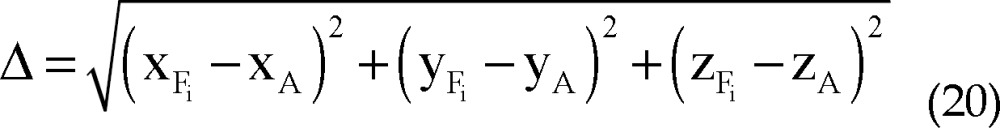


To determine the significance of the difference between the anatomical and functional targets along the three axes (X, Y, and Z) an analysis was carried out using SPSS software (version 16, SPSS Inc., Chicago, IL, USA). We used Wilcoxon and paired t-tests for analyzing this difference. Pearson’s correlation coefficient defined the strength of linear dependence between coordinates of two targets on each axes for comparing accuracy of targeting between different methods. Furthermore, independent t-test and analysis of variance (ANOVA) compared the “delta” between right and left sides on the five different trajectories (anterior, central, medial, lateral and posterior).

## Results

**Figure 4:**
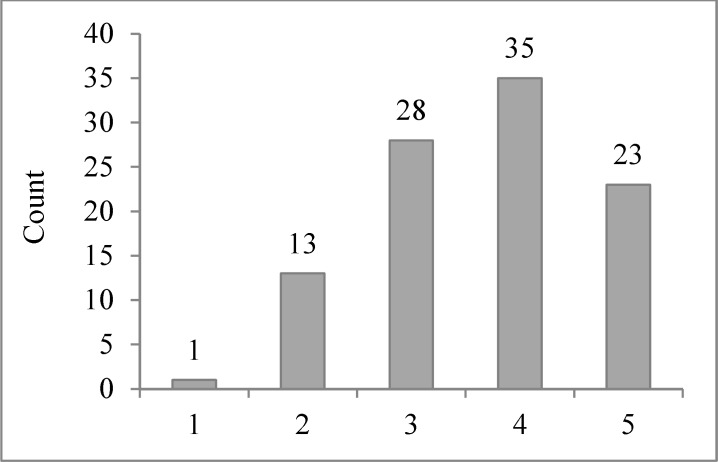
Number of trajectories in which subthalamic nucleus signal was recorded

Of the 50 bilaterally implanted patients in this study, 9 were females and 41 were males. The mean age was 50.45 ± 9.17 years. We recorded STN signals in 1 and 5 trajectories in 1 and 22% of STNs respectively (Figure 4). Table 2 shows the mean lengths of STN recorded on various trajectories on the right and left sides.

**Figure 5:**
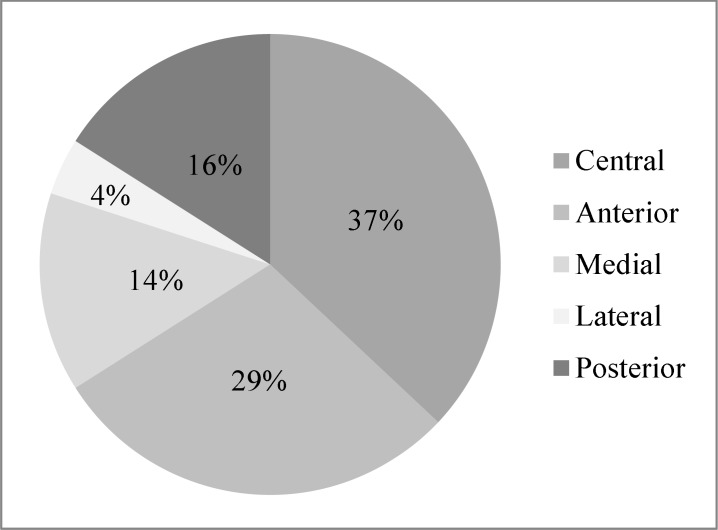
Trajectory selection distribution

In each case, we selected one of the five trajectories (anterior, central, medial, lateral or posterior) for placement of the permanent DBS lead on either sides and most frequently it was the central trajectory (Figure 5).

We determined the anatomical target (by six different methods) and the functional target along the three axes(X, Y, and Z). We evaluated the normal distribution of the parameters with one-sample Kolmogorov-Smirnov along each coordinate axes. Parameters of Y and Z coordinates had normal distributions and for X coordinate, the distribution was not normal.

For the X coordinate, using non-parametric Wilcoxon test, we compared the statistical difference between anatomical and the functional targeting methods. Only in the atlas-based method, we found a statistical difference between them along the X axis (Table 3).

**Table 2:** Mean lengths of subthalamic nucleus recorded on various trajectories on right and left sides

**Trajectory (mm)**	**Central**	**Anterior**	**Posterior**	**Medial**	**Lateral**
**Target**					
Right STN (mean length ± SD )	4.02 ± 2.30	3.10 ± 2.72	3.44 ± 2.16	3.16 ± 2.34	2.43 ± 2.53
Left STN (mean length ± SD )	4.20 ± 2.06	3.61 ± 2.81	2.65 ± 2.42	2.08 ± 2.18	2.42 ±2.22

SD: Standard deviation; STN: Subthalamic nucleus

Using paired t-test, there was a statistical difference between functional and all six anatomical targeting methods on Y axis; but on the Z axis, just in the manual/axial plane we found a statistically significant difference (Table 2).

**Figure 6:**
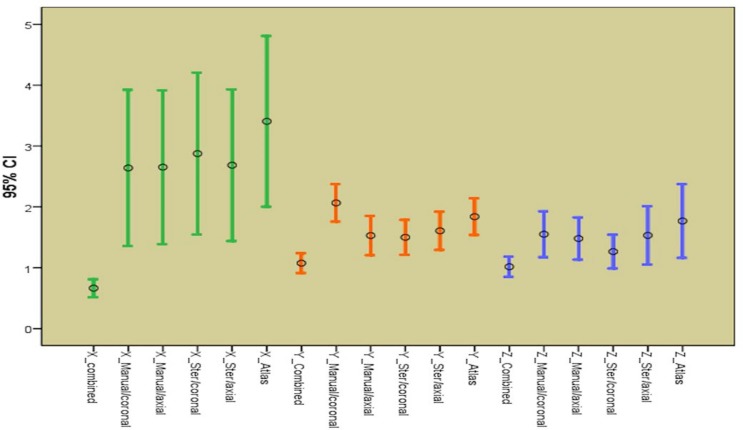
Comparing distances between anatomical and functional targets

Comparing the distances between functional and anatomical targets in six methods on three coordinates which can show the accuracy and partial error of targeting between these anatomical methods (Table 4 and Figure 6).

The less the mean distance and the range of confidence interval, the more accurate is the targeting method, so we also defined the correlation between coordinates of functional and anatomical targets in six methods on each axis (Table 5).

**Table 3:** Significance of difference between position of functional and anatomical targets on X, Y and Z coordinate axes

**Coordinate axis**		**Combined**	**Manual ** **coronal**	**Manual ** **axial**	**Stereonauta ** **coronal**	**Stereonauta ** **axial**	**Atlas ** **based**
Significance (two-tailed)	X (Wilcoxon)	0.950	0.340	0.190	0.580	0.720	0.047
	Y (paired t-test)	0.010	< 0.001	0.037	0.002	< 0.001	0.001
	Z (paired t-test)	0.710	0.180	0.040	0.920	0.290	0.650

**Table 4:** Distance between functional and anatomical targets in six different methods on X, Y, and Z coordinate axes

**Coordinate axes**	**Combined**	**Manual ** **coronal**	**Manual ** **axial**	**Stereonauta ** **coronal**	**Stereonauta ** **axial**	**Atlas ** **based**
X						
Mean	0.66	2.63	2.65	2.87	2.68	3.4
Range of 95% CI	0.51-0.81	1.35-3.9	1.39-3.9	1.54-4.2	1.4-3.9	2-4.8
SE	0.07	0.64	0.63	0.66	0.62	0.7
Y						
Mean	1.07	2.06	1.52	1.50	1.60	1.84
Range of 95% CI	0.91-1.23	1.75-2.37	1.2-18	1.21-1.78	1.29-1.92	1.53-2.1
SE	0.08	0.15	0.16	0.14	0.15	0.15
Z						
Mean	1.01	1.55	1.48	1.26	1.53	1.76
Range of 95% CI	0.85-1.18	1.17-1.92	1.13-1.82	0.98-1.54	1.05-2.01	1.16-2.37
SE	0.08	0.18	0.17	0.13	0.24	0.30

SE: Standard error; CI: Confidence interval

The mean difference between combined method of anatomical targeting and functional target chosen is defined as “delta” was 1.84 ± 0.88 mm (range: 0-4.25 mm). Using the independent t-test, we compared delta on the right and left sides (1.83 ± 0.91 and 1.75 ± 0.86 mm, respectively) and found no statistical difference between them (P = 0.680).

Identifying delta for each trajectory separately (Table 6), the central trajectory was the least (0.98 ± 0.74 mm) with an interesting statistical difference with others (using least significant difference-post-hoc test of ANOVA, P < 0.001).

The pre-operative unified PD rating scale III scores OFF and ON medication was 54.52 ± 5.40 and 18.22 ± 2.88, respectively. Post-operative score yielded was 12.80 ± 3.14 in stimulation ON and medication ON (with a 40% decrease in L-dopa equivalent dosage) state that showed significant difference comparing with both pre-operative scores (P < 0.001).

This study was conducted to evaluate the accuracy and precision of six anatomical targeting methods in comparison with intra-operative localization using MER and MES. This is arranged according to Pearson’s correlation coefficient between coordinates of two targets along each axes in table 7.

**Table 5:** Pearson’s correlation coefficient between coordinates of two targets on each axes for six different methods

**Axes** **‎**	**Combined**	**Manual coronal**	**Manual axial**	**Stereonauta coronal**	**Stereonauta axial**	**Atlas based**
X	0.990	0.790	0.800	0.780	0.82	0.760
Y	0.920	0.814	0.751	0.792	0.818	0.775
Z	0.970	0.820	0.839	0.857	0.856	0.775

**Table 6:** Descriptive analysis of “delta” on five different trajectories

**Electrode**	**Mean ± SD**	**SE**	**95% CI** **‎** ** for mean**		**Minimum**	**Maximum**
			**Lower bound**	**Upper bound**		
Posterior	2.551 ± 0.629	0.157	2.216	2.886	2.00	4.25
Central	0.986 ± 0.743	0.122	0.739	1.234	0.00	2.50
Anterior	2.292 ± 0.322	0.060	2.169	2.415	2.00	3.40
Medial	2.315 ± 0.491	0.131	2.032	2.599	2.00	3.61
Lateral	2.160 ± 0.228	0.114	1.797	2.522	2.02	2.50
Total	1.848 ± 0.879	0.088	1.674	2.023	0.00	4.25

SD: Standard deviation; CI: Confidence interval; SE: Standard error

**Table 7:** Relative accuracy of six targeting methods on each axis

**Different ** **‎** **methods**	**X**	**Y**	**Z**
The most accurate	Combined	Combined	Combined
2^nd^	Stereonauta/axial	Stereonauta/axial	Stereonauta/coronal
3^rd^	Manual/axial	Manual/coronal	Stereonauta/axial
4^th^	Manual/coronal	Stereonauta/coronal	Manual/axial
5^th^	Stereonauta/coronal	Atlas based	Manual/coronal
The least accurate	Atlas based	Manual/axial	Atlas based

## Discussion

The most accurate targeting method on each coordinate axes was a combination of all six methods by an experienced functional stereotactic neurosurgeon and the second most accurate method was using the Stereonauta software in coronal plane for Z and in axial plane for Y and X coordinates. Overall the combination of all methods is the closet anatomical estimate of the physiological target. The spatial position of the functional target is calculated later in this section. 

Several prior studies using atlases for targeting are known to be not accurate enough and have remarkable limitations, as they are extracted from limited brain specimens and also with degeneration antero-supero-lateral transposition of the STN occurs with aging chronologically and in the disease life cycle itself.^11^^,^^15^^-^^17^

On the other hand, in a study performed by Zonenshayn et al.^18^ to compare different anatomical targeting methods, the most precise was a combined approach and followed by an mid-commissural point based method using the Schaltenbrand and Wahren Atlas. Interestingly MRI-guided targeting had the least accuracy.

In the current study, anatomical atlas-based indirect targeting presented among the two least accurate methods on three axes. Direct targeting using T2 MRI was the second most accurate method. 

In some other studies, MER and MES were reported to be time-consuming procedures, which were associated with complications such as ICH and infection. They were not helpful in improving accuracy of targeting either.^3^^,^^6^

Foltynie et al.^19^ studied different targeting methods on 79 patients and emphasized that the ideal method remains unknown but MER may lead to increased complications and even death. They attribute increase in surgery time, dural opening and consequently prolonged cerebrospinal fluid leakage may itself exacerbate brain shift; however precise anatomical targeting without dural opening is sufficient to obtain optimal results.

In the current study, the time required for five simultaneous MER trajectories and subsequent MES per STN was about 60 minutes. In a 1-year follow-up after surgery, no superficial or deep infection was observed. During the procedure, a semi-sitting position was used (as close as possible to the MRI acquisition position), placement of the frontal burr holes on the uppermost part of the skull and also continuous irrigation with normal saline minimized intracranial air penetration and possible brain shift.^17^

In addition, it merits consideration that comparison of anatomical-functional target distances on each coordinate axes revealed statistical difference on Y coordinate between all six pre-operative localization methods and on X and Z coordinates, in Atlas and manual/axial methods respectively.

It is worthwhile noting that among our 100 STN DBS procedures, non-central trajectories were chosen for permanent stimulation as the functional target in 63%. Thus, although accurate pre-operative anatomical targeting may reduce the need for invasive intra-operative exploration and thereby decrease the surgical duration and procedure-related complications, certain intra-operative electrophysiological measurements are still required to compensate for the possible inadequacy of these targeting methods.

A review article, which was published by Benabid,^20^ emphasizes on efficacy of MER despite the presence of some potential complications. In a study by Molinuevo et al.^3^, on 15 PD patients who underwent bilateral STN DBS, a significant difference between location of pre-operative anatomical target and final surgical target was found (2.1 ± 1.3 mm, more than 4 mm in 10% of patients). In the current study, the difference between these two targets in 100 procedures was 1.84 ± 0.88 mm (more than 3 mm in 7% of cases).

In the current study, we determined the mean “delta” for each trajectory and found a significant difference between the central trajectory (0.99 mm) and other trajectories which might be due to a measurement bias. In fact, “delta” on the central trajectory could be any value but the least it could be on other trajectories is 2 mm, as the distance between central and other four parallel trajectories is 2 mm.

Many previously published studies emphasize on beginning the procedure on the contralateral side of the patient’s dominant symptoms because between the two STN (left and right) procedures, targeting accuracy may significantly decrease on the second side due to brain shift and even perform more comprehensive MER on this side.^21^ Despite beginning the surgical procedure on the left side in all patients at our center, we found no statistically significant difference between “delta” on both sides which were 1.75 ± 0.86 and 1.83 ± 0.92 mm on right and left STN’s respectively (P = 0.680).

Finally, we used the following formulae as a mathematical model for composition of inaccuracy of anatomical targeting and probable intra-operative brain shift to identify X, Y, and Z coordinates of the final (functional) target for the right/left STN according to the coordinates of the anatomical target, depth at which the permanent DBS lead was placed (distal end of the distal most electrode) and arc and ring angles on the Leksell stereotactic system.

Right lateral trajectory and left medial trajectory:


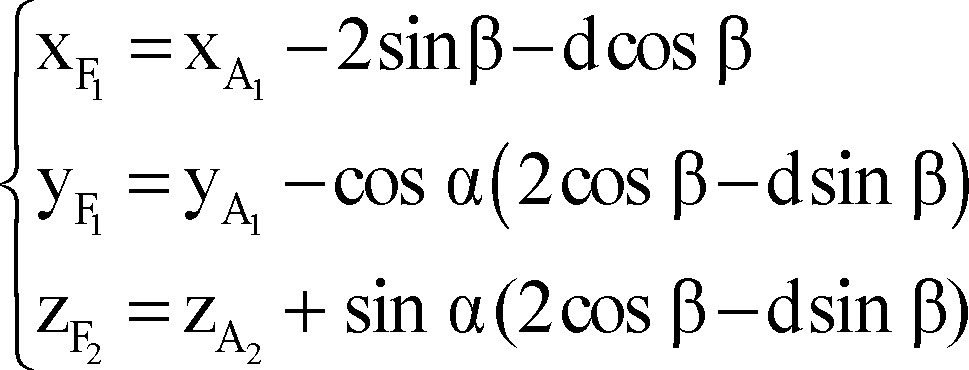


Right medial trajectory and left lateral trajectory:


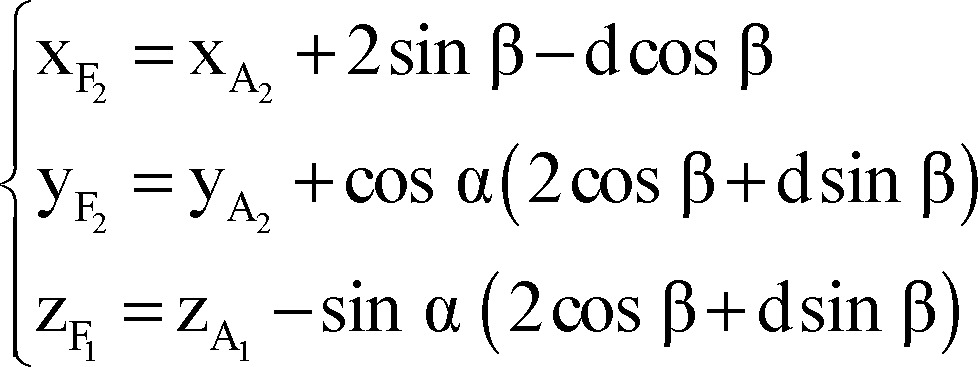


Right or left anterior trajectory:


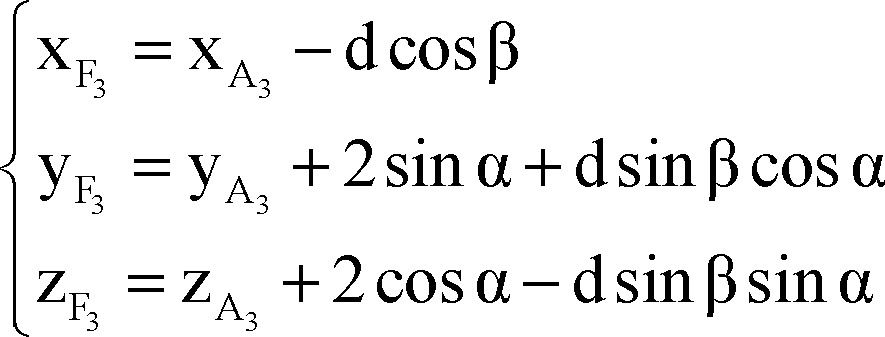


Right or left posterior trajectory:


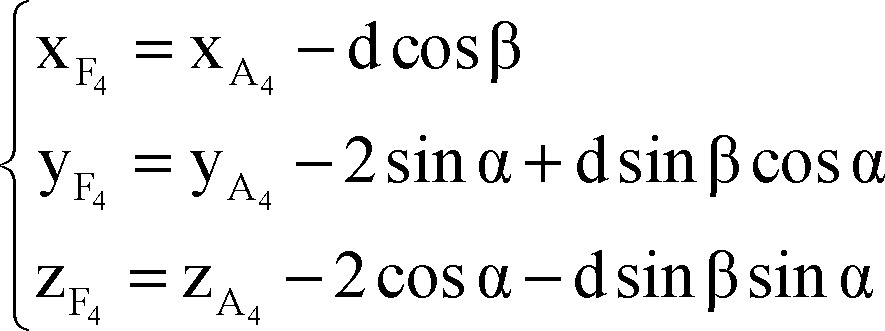


Right or left central trajectory:


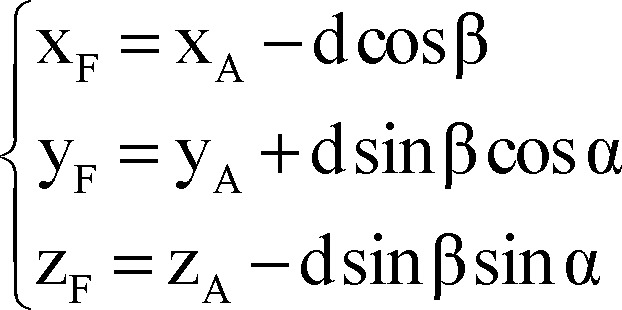


We could not identify any formulae for calculating these parameters according to pre- and intra-operative findings during the literature review. These could easily be incorporated in a simple program like Microsoft Excel (version 2010). In studies that used post-operative MRI for determining the exact location of permanent electrode to compare it with pre-operative target, magnetic artifact of the electrode itself, represented as the origin of errors in this process.^22^^-^^25^

Limitations in the above study can be addressed in future research. It would have been more purposeful to perform a post-operative conditionally safe MRI brain scan to compare the anatomical location with the final lead location as guided by the intra-operative electrophysiology. This would be relevant to identify systematic errors that can be corrected by a dynamic correction factor which can be used to recalculate the stereotactic coordinates.

## Conclusion

The most physiologically accurate method for anatomical targeting is a combination of multiple independent methods with experience of a stereotactic neurosurgeon and it is ideal to refine the same with intra-operative neurophysiological recording (MER) and stimulation (MES) to identify the optimal functional target in real time.

A question to be answered in future studies is the significance of a number of recording trajectories (single sequential vs. multiple simultaneous) and whether a correlation is present between intra-operative neurophysiological monitoring and better clinical outcome?

## Notes:

**How to cite this article:** Parvaresh-Rizi M, Tabibkhoei A, Shahidi Gh, Vaidyanathan J, Tabibkhoei A, Rohani M. Spatial distance between anatomically- and physiologically-identified targets in subthalamic nucleus deep brain stimulation in Parkinson’s disease. Iran J Neurol 2016; 15(1): 34-45.
